# Adipokine human Resistin promotes obesity-associated inflammatory intervertebral disc degeneration via pro-inflammatory cytokine cascade activation

**DOI:** 10.1038/s41598-022-12793-2

**Published:** 2022-05-27

**Authors:** Jae Hee Shin, SeongHyun Park, Hansang Cho, Joo Han Kim, Hyuk Choi

**Affiliations:** 1 Department of Medical Sciences, Graduate School of Medicine, Korea University, 80, Guro-dong, Guro-gu, Seoul, 152-703 South Korea; 2 Department of Electrical and Electronic Engineering, The University of Manchester, Manchester, UK; 3 Department of Biophysics, Sungkyunkwan University, Suwon, South Korea; 4 Department of Neurosurgery, Guro Hospital, College of Medicine, Korea University, Seoul, South Korea; 5 Institute of Quantum Biophysics, Sungkyunkwan University, Suwon, South Korea; 6 Intelligent Precision Healthcare Convergence, Sungkyunkwan University, Suwon, South Korea

**Keywords:** Cytokines, Enzymes

## Abstract

Adipokine human Resistin (hResistin), is known to be associated with insulin resistance and secrete low-grade pro-inflammatory cytokines in obesity. Although studies on low-grade inflammation of adipokine hResistin are known, studies on the effects and mechanisms of intervertebral disc degeneration (IVDD) are still lacking. Thus, we investigated the adipokine hResistin with or without pro-inflammatory cytokine IL-1β in intervertebral disc (IVD) cells such as human annulus fibrosus (hAF) and nucleus pulposus (hNP). The protein expression changes in IL-1β, IL-6, IL-8, MMP-1, MMP-3, and MMP-13, induced by the combined-hResistin and IL-1β stimulation on hAF cells, was significantly greater than that of the same induced by mono-IL-1β stimulation. Similarly, in the case of the protein expression change of inflammatory mediators induced by the combined-hResistin and IL-1β stimulation on hNP cells was also significantly greater than that of the same induced by mono-IL-1β stimulation. These results improve understanding of hResistin on inflammatory IVDD but also with other obesity-related inflammatory diseases.

## Introduction

In extension of the discovery of the low-grade pro-inflammatory cytokine activation nature of adipokines via the enhancement of secretion of pro-inflammatory mediators such as tumor necrosis factor (TNF)-α and interleukin (IL) families, which have been widely reported to be associated in the development of insulin resistance in obesity, understanding the mechanism of development and progression of the obesity-associated inflammatory intervertebral disc degeneration (IVDD) has received a strong attention to pave the way towards the development of better preventative and treatment measures for obesity-related degenerative disc diseases^1–3^. Among various adipokines, leptin has been widely studied to investigate pathogenic mechanism of obesity-related inflammatory IVDD^1,4–7^. However, in contrast, pathogenic mechanism of obesity-related inflammatory IVDD-associated with other adipokines such as resistin has not been widely investigated. In 2016 Liu et al. reported the upregulation of expression of a disintegrin and metalloproteinase with thrombospondin motifs (ADAMTS)-5 via activation of p38 MAPK pathway induced by exposure of Resistin on rat nucleus pulposus (NP) cells^8^. ADAMTS-5 is widely reported to contribute IVDD^9^. In the following year, 2017, Li et al. reported a significant increase of expression of one of the macrophage inflammatory protein, CCL4, via activation of p38 mitogen-activated protein kinase (MAPK) and nuclear factor kappa B (NF-κB) signaling pathways induced by exposure of hResistin on hNP cells^10^. Meanwhile, IVD cells can produce IL-1β like immune cells, and recently, along with the studies on the IVDD mechanism, activation of nod-like receptor protein 3 (NLRP3) inflammasome has been linked to disc inflammation, pyroptosis, extracellular matrix (ECM) degradation, and apoptosis of IVD cells^11,12^. Also in another study, pathogen infection of hNP cells confirmed pyroptosis through NLRP3 activation^13^. Furthermore, it was reported that the expression of NLRP3, caspase-1, and IL-1 was upregulated in IVDD tissue compared to normal disc tissue, which correlated significantly with NLRP3^14^. However, IVD is composed of avascular tissue except for the outer AF area, and nutrients that necessary for cell growth and survival diffuse through the vertebral blood vessels from the outer AF to the NP area^15,16^. Resistin is mainly produced in mouse adipose tissue and is known to affect pro-inflammation. In humans, Resistin is mainly produced in macrophages, and macrophage infiltration is found in degenerative disc tissue accompanied by inflammation^17^. Therefore, we hypothesized that Resistin would act on the intervertebral disc and promotes the inflammatory response in inflammatory disc degenerative disease. And to the best of our knowledge, the correlation between the low-grade inflammation inducing nature of hResistin and the progression of inflammatory IVDD, which is associated with the promotion of various pro-inflammatory cytokine cascade activation, has not been previously reported. Additionally, it is important to investigate hResistin effects on hIVD cells to determine the pathomechanism of early-stage IVDD in obesity.

The objective of the present study was to investigate the effects of hResistin on hIVD cells in inflammatory cytokines, inflammasome, and ECM degradation in IVDD. Our results suggests that hResistin upregulate inflammatory mediators, such as NLRP3 inflammasome, pro-IL-1β, IL-6, and IL-8, and ECM-catabolic enzymes expression, such as matrix metalloprotease (MMP)-1, MMP-3, and MMP-13 through the nuclear factor kappa B (NF-κB) and mitogen-activated protein kinase (MAPK) signaling pathway, which are initial events in IVDD progression. Hence, in this study, the promoting effect of hResistin on the promotion of progression of inflammatory disc degeneration-associated IL-1β pro-inflammatory cytokine cascade activation has been investigated.

## Materials and methods

### Isolation and culture of hIVD cells

Human IVD cells were isolated from disc tissues of six patients (four males and two females) with degenerative spinal disease (Pfirrmann degenerative grades II–III) during elective surgery. Disc tissue collection was conducted with the approval of the institutional review board (KUGH170208-001) of Korea University Hospital, and informed consent was obtained from the subjects. All methods were performed in accordance with the guidelines and regulation of the Korea University Hospital Human Ethics Committee. The intervertebral disc tissue obtained from the patient was separated into AF with a dense and elastic fibrous structure and NP with a white, soft and loose appearance. Tissue specimens were placed in Ham’s F-12 medium (Gibco-BRL, Grand Island, NY), containing 5% fetal bovine serum (FBS; Gibco-BRL) and 1% penicillin/streptomycin (P/S; Gibco-BRL), and then washed with phosphate-buffered saline (PBS; Gibco-BRL). Both annulus fibrosus (AF) and NP sections were minced and digested for 1 h at 37 ℃ in an incubator under gentle stirring in F-12 medium containing 5% FBS, 1% P/S, and 0.2% pronase (Calbiochem, La Jolla, CA, USA). Subsequently, the minced AF and NP tissues were incubated overnight with 0.025% collagenase I (Roche Diagnostics, Mannheim, Germany). After overnight incubation, the cells were filtered through a sterile nylon mesh (70-µm pore size) and centrifuged (676×*g*, 5 min). Both AF and NP pellets were resuspended in F-12 medium supplemented with 10% FBS and 1% P/S, and then cultured at 37 °C in a humidified atmosphere containing 5% CO_2_.

### Cell viability assay (WST-1 assay)

Followed by the growth of IVD cells preparing to be confluent, the subsequent overnight serum starvation using 1 μg/mL of mitomycin C reagent was applied to inhibit progression of cell cycle. The cell viability of hIVD was measured by WST-1 colorimetric assay (ez-1000; Daeillab) followed by stimulation of hResistin (Peprotech) at five-different concentrations such as 25, 50, 100, 200, and 400 ng/mL for 48 h. Subsequently 10 µL of WST-1 reagent was added into the resultant hResistin stimulated samples, which were seeded into a 96-well plate at a concentration of 3 × 10^3^/well in 200 µL culture medium, followed by incubation for 4 h at 37 °C. Lastly, the absorbance values of the incubated samples were recorded at 450 nm using the spectrophotometer.

### Cell cytotoxicity assay (LDH and live/dead assay)

To evaluate the possible cytotoxicity effects, cell cytotoxicity was verified with respect to different concentrations (0, 25, 50, 100, 200, and 400 ng/mL) of hResistin using lactate dehydrogenase (LDH; Roche) and Live/Dead assay (Calcein-AM and Ethidium Homodimer-1; Invitrogen). 5 × 10^4^ of both hAF and hNP cells were seeded in a 35-mm culture dish (SPL) with subsequent mono-hResistin stimulation. The culture supernatant (conditioned medium; CM) was collected after 48 h for LDH assay. And then, adherent cells were treated with Calcein-AM (stained live cells) and Ethidium Homodimer-1 (stained dead cells) according to the manufacturer’s procedure to identify live cells and dead cells.

### Stimulation of reagents on hIVD cells

**Figure 1:**
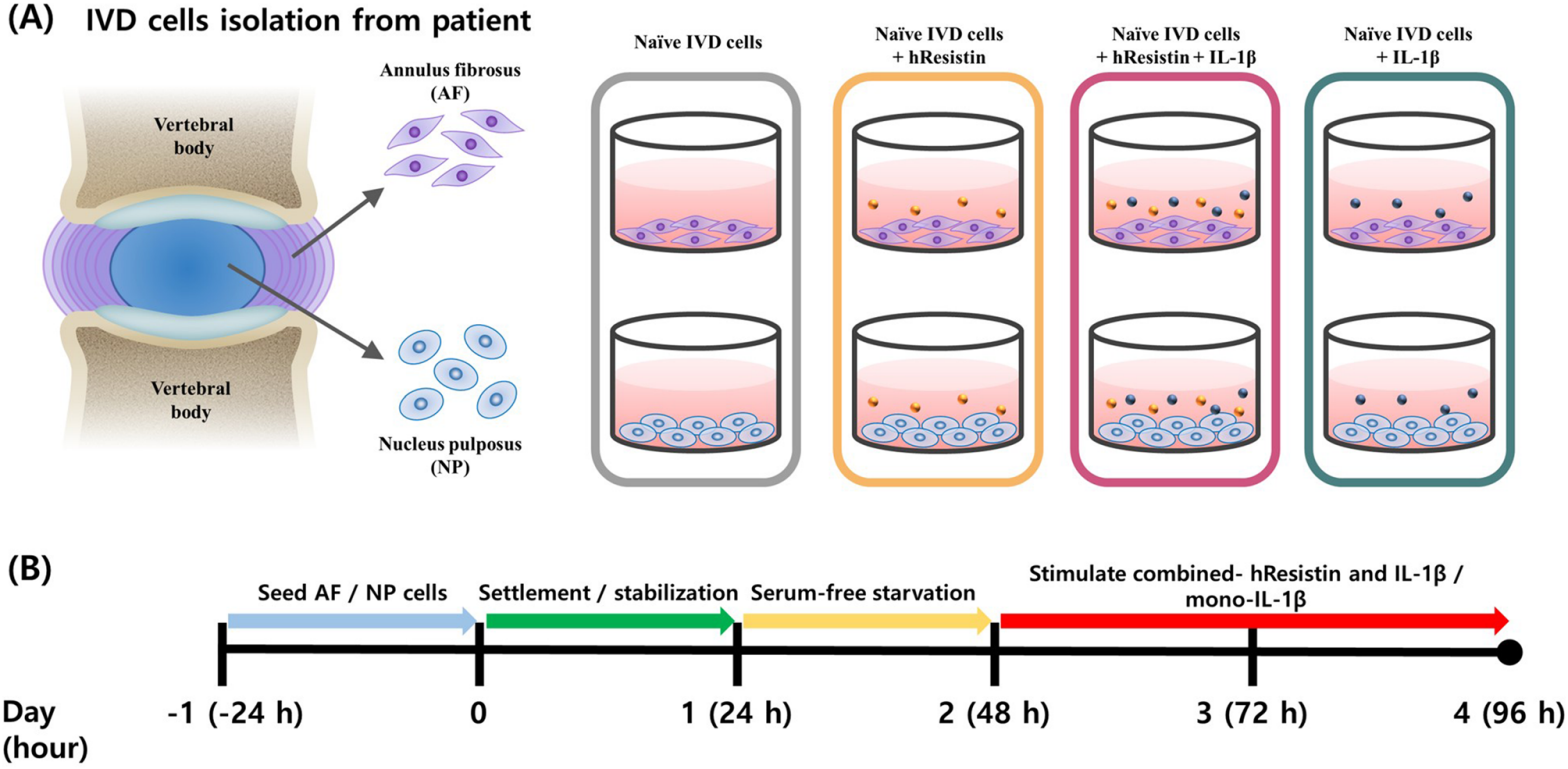
(**A**) Schematic design and (**B**) timeline of the experiment used for the mono-hResistin, combined-hResistin and IL-1β or mono-IL-1β stimulation of hAF and hNP.

The cultured hAF and hNP cells were subjected to stimulation of mono-hResistin (100 ng/mL), combined-hResistin (100 ng/mL) and IL-1β (1 ng/mL) or mono-IL-1β (1 ng/mL) for 48 h. Prior to the stimulation process, the both hAF and hNP cells, seeded in six-well plates with stabilization for 24 h, were undergone serum starvation for 24 h. The schematic design and timeline of experiment for stimulation process is shown in Fig. 1.

### Real-time qRT-PCR

Total RNA was extracted using TRIzol reagent (Invitrogen, Carlsbad, CA, USA). The quantity and quality of the extracted RNA were determined using a Nanodrop 2000 Spectrophotometer (Thermo Scientific, MA, USA). RNA (2000 ng) was used to synthesize cDNA using a reverse transcription kit (Toyobo FSQ 101; Toyobo Co., Ltd., Osaka, Japan), in accordance with the manufacturer’s protocol. Real-time PCR amplification was performed as follows: 40 cycles at 95 °C for 15 s for denaturation and 60 °C for 60 s for annealing and elongation. Then, performed for pro-IL-1β, Caspase-1, and NLRP3 using the SYBR Green PCR Master mix (Applied Biosystems, MA, USA). The primer sequences used were as follows: pro-IL-1β, forward: 5′-TTCGACACATGGGATAACGAGG-3′; reverse: 5′-TTTTTGCTGTGAGTCCCGGAG-3′; Caspase-1, forward: 5′-TTTCCGCAAGGTTCGATTTTCA-3′; reverse: 5′-GGCATCTGCGCTCTACCATC-3′; NLRP3: forward: 5′-CCACAAGATCGTGAGAAAACCC-3′; reverse: 5′-CGGTCCTATGTGCTCGTCA-3′; GAPDH: forward: 5′-CTGGGCTACACTGAGCACC-3′; reverse: 5′-AAGTGGTCGTTGAGGGCAATG-3′. Gene expression was analyzed using the 2^−ΔΔCt^ method and normalized to that of housekeeping gene GAPDH. Naïve hIVD cells were used as controls for hResistin stimulated hIVD cells.

### Western blot analysis

hAF and hNP cells lysates were prepared using RIPA buffer (Biosesang, Seongnam, Korea) with protease inhibitor such as 1 M of PMSF (Roche, BS, Switzerland) and phosphatase inhibitor such as 0.2 mM of Na_3_VO_4_ (Sigma, MO, USA) and 10 mM of NaF (Sigma). Extracted total protein was evaluated using a bicinchoninic acid (BCA) assay. A sodium dodecyl sulfate (SDS) poly-acrylamide gel (10%) was loaded with 40 µg of total protein, followed by electrophoresis (SDS-PAGE). The resolved proteins were then transferred to polyvinylidene fluoride (PVDF) membranes (Bio-Rad, CA, USA), which were subsequently blocked with 5% skim milk or 3% BSA and incubated overnight at 4 °C with 5% BSA in TBST (Tris-buffered saline with 0.1% Tween 20) with the primary antibody. Immunolabeling was detected using ECL reagent (Invitrogen, MA, USA). The antibodies used for analysis were anti-phospho-p65, anti-p65, and anti-β-actin antibodies, which were purchased from Santa Cruz Biotechnology (1:1000; Dallas, TX, USA), and anti-phospho-p38 and anti-p38 antibodies were purchased from Cell Signaling Technology (1:1000; Danvers, MA, USA).

### Enzyme-linked immunosorbent assay (ELISA)

The concentration of inflammatory cytokines (IL-1β, IL-6, and IL-8) and extracellular matrix (ECM)-catabolic enzymes (MMP-1, MMP-3, and MMP-13) in the CM, induced by 48 h of combined-hResistin and IL-1β stimulation or mono-IL-1β, was determined by using the commercially available ELISA kits (R&D Systems, MN, USA) in accordance with the manufacturer’s procedure. The ELISA results used the standard curve of the optical density values of the standard antibodies to derive a linear regression equation and then calculate the corresponding concentration of the unknown samples.

### Treatment of hIVD cells with IL-1 receptor, p38 MAPK, and p65 NF-κB-specific inhibitors, followed by combined-hResistin and IL-1β stimulation

hAF and hNP cells were cultured in 6-well plates and incubated at 37 ℃ with the IL-1 receptor antagonist IL-1Ra (100 ng/mL), the p38 inhibitor SB203580 (10 uM, Sigma Chemical Co., St. Louis, MO, USA), and IκB kinase inhibitor Bay 11-7082 (5 mM, Sigma) at the indicated concentrations for 1 h before combined-hResistin and IL-1β stimulation.

### Statistical analysis

To obtain the mean ± standard deviation of the analysis results of individual cells, six experiments with stimulation of combined-hResistin and IL-1β or mono-IL-1β were conducted. The obtained results were subjected to the One-way analysis of variance (ANOVA) with Bonferroni correction post hoc test. The normal distribution of each subgroup was evaluated by the Shapiro–Wilk test. For data that do not exhibit a normal distribution, we used Kruskal–Wallis with Dunn’s multiple comparison test. Differences with p < 0.05 were assumed to be statistically significant. All statistical analyses were performed using SPSS software (ver. 21.3, SPSS Inc., Chicago, IL, USA).

## Results

### Determination of optimum concentration of hResistin stimulation on hIVD cells in vitro

**Figure 2:**
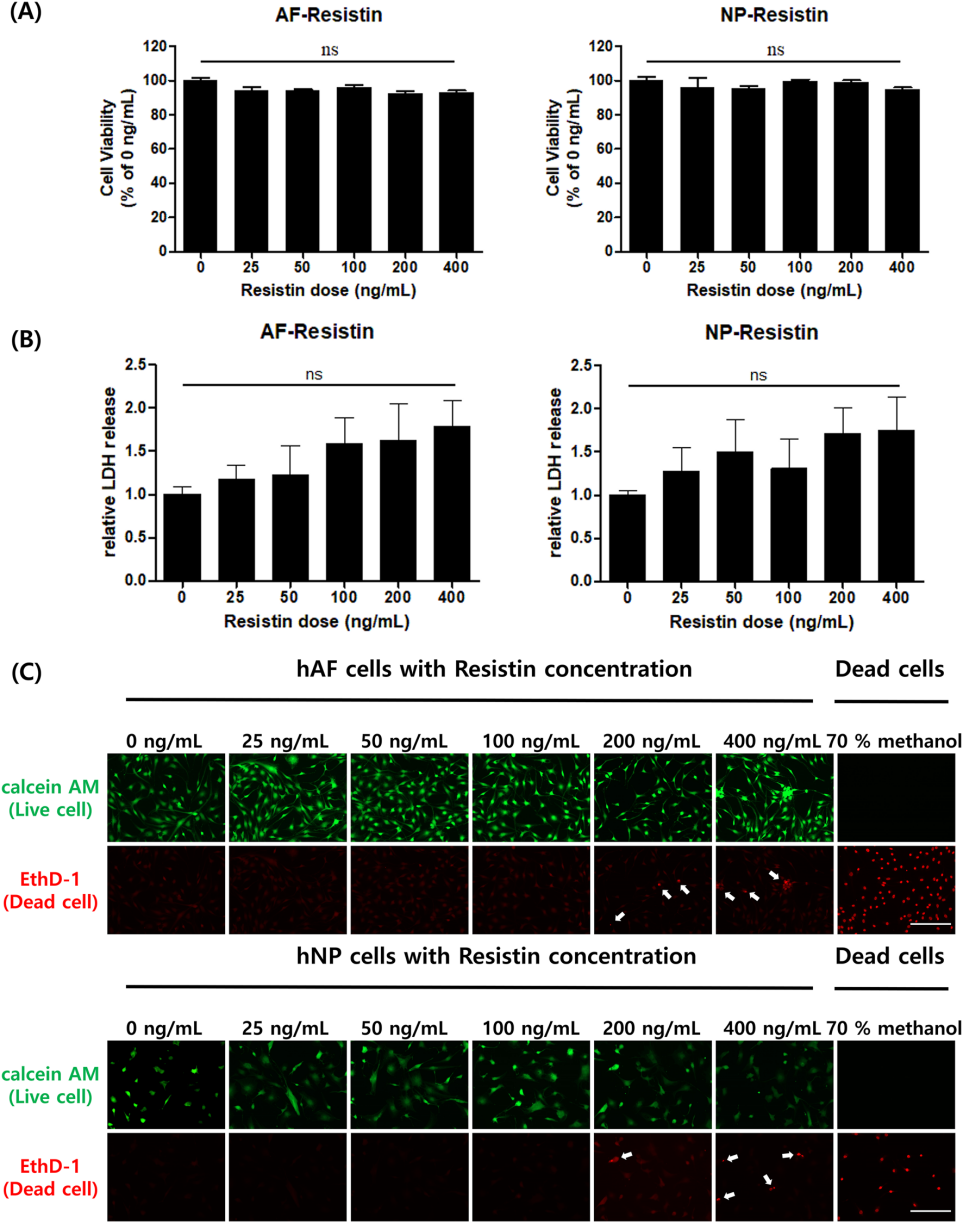
The mean ± SD of (**A**) viability, (**B**) cytotoxicity, and (**C**) fluorescence image (scale bar = 200 µm) of the live/dead assay of hIVD cells stimulated with six different concentrations of hResistin (0, 25, 50, 100, 200 and 400 ng/mL, n = 5/group) in vitro.

As shown in Fig. 2, in order to investigate the optimal concentration of hResistin treatment on the cultured hAF and hNP cells in vitro, the viability, cytotoxicity and fluorescence image of cytotoxicity of the pre-cultured hAF and hNP cells with 6 different concentrations of hResistin treatment, which are 0, 25, 50, 100, 200, and 400 ng/mL, were measured by using WST-1, LDH, and live/dead assay, respectively. The viability and cytotoxicity were measured in mean ± SEM, whereas the fluorescence image of cytotoxicity was shown in 200 µm scale bar. Based on the resultant fluorescence image of cytotoxicity shown in Fig. 2C, 100 ng/mL of hResistin was selected as an optimal concentration in this study since the dead hAF and hNP cells began to be observed in the group with 200 ng/mL of hResistin stimulation. This can be also supported by the non-significantly increased of secreted LDH level of hAF and hNP cells as shown in Fig. 2B, without a significant decrease of their viability of the same group as shown in Fig. 2A, with respect to that of the naïve group.

### Correlation between hResistin stimulation and phosphorylation of p38 MAPK and p65 NF-κB

**Figure 3:**
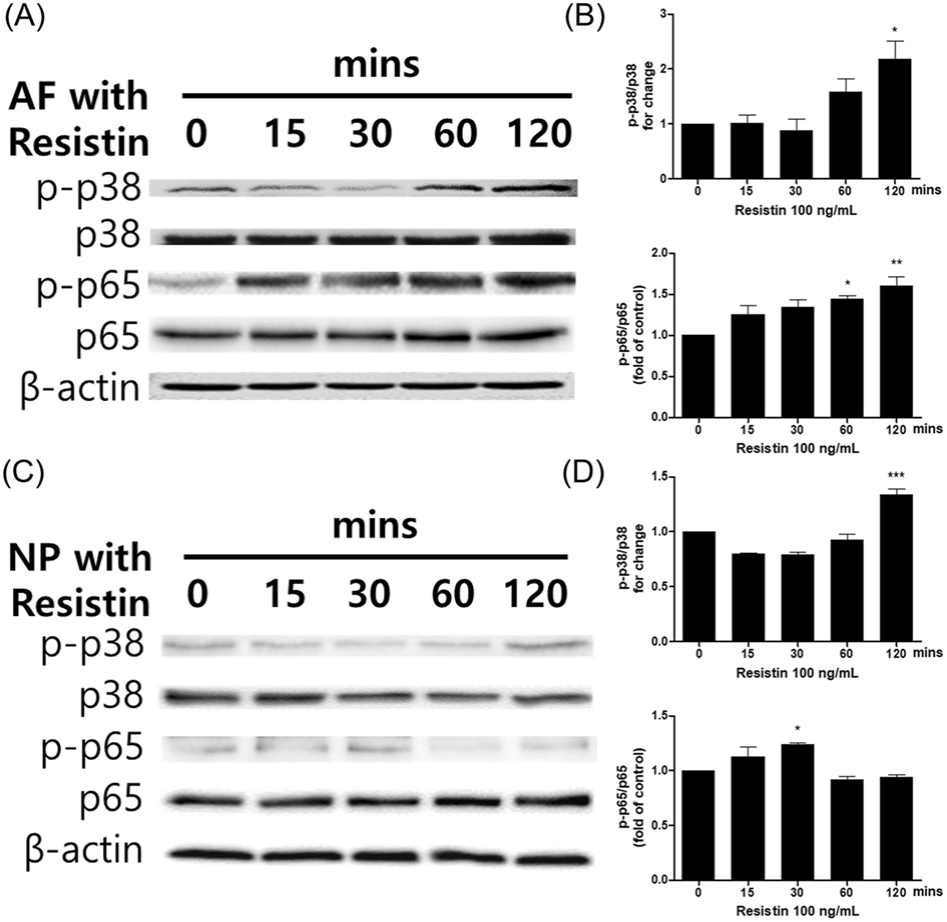
(**A,C**) The signaling pathway and (**B,D**) the densitometric quantification (*p < 0.05, **p < 0.01, and ***p < 0.001) of phosphorylated p38 MAPK and p65 NF-κB induced by 5 different durations of post-hResistin stimulation, compared to the unstimulated naïve control group (n = 5/group).

As shown in Fig. 3 and Supplementary Fig. S1, phosphorylation of p38 MAPK and p65 NF-κB induced by five-different durations of hResistin stimulation (0, 15, 30, 60 and 120 min) on hAF and hNP cells was quantified. hAF showed 2.18-fold (p < 0.05) of p38 MAPK protein phosphorylation and 1.6-fold (p < 0.01) of p65 NF-κB protein phosphorylation at 120 min timepoint compared to the control group. In the case of hNP, protein phosphorylation of p38 MAPK was 1.33-fold (p < 0.001) at 120 min, and protein phosphorylation of p65 NF-kB was 1.24-fold (p < 0.05) at 30 min.

### Gene expression of inflammasome components

**Figure 4:**
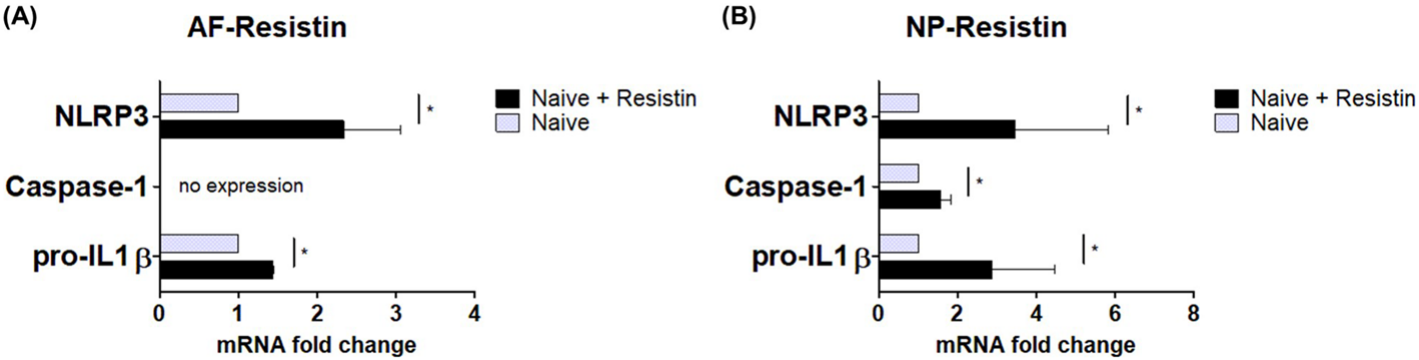
mRNA gene expression of the inflammatory mediators of NLRP3, Caspase-1, and pro-IL1β between IVD cells and hResistin stimulation, compared to unstimulated naïve IVD control groups (*p < 0.05, n = 5/group).

The mRNA expression of inflammasome components, such as nod-like receptor protein 3 (NLRP3), Caspase-1, and pro-IL-1β, was induced by hResistin stimulation, and the results are shown in Fig. 4. The relative fold-changes of NLRP3 and pro-IL1β gene expression compared to those in the unstimulated and control groups were 2.87-fold (p < 0.05), 1.44-fold (p < 0.05) in hAF cells. Additionally, mRNA expression of caspase-1 was not observed. In the case of hNP cells, the relative fold-changes of NLRP3 and pro-IL1β gene expression compared to those in the unstimulated and control groups were 3.47-fold (p < 0.05), 1.56-fold (p < 0.05), and 2.87-fold (p < 0.05), respectively.

### The role of stimulation of hResistin on the promotion of IL-1β-associated pro-inflammatory cytokine cascade activation

Relative fold changes of the inflammatory mediators secretion of IL-1β, IL-6, IL-8, MMP-1, MMP-3, and MMP-13 proteins in the combined-hResistin and IL-1β stimulation groups compared to the mono-IL-1β groups in hAF were 3.50-fold (p < 0.001), 9.75-fold (p < 0.001), 1.77-fold (p < 0.01), 3.77-fold (p < 0.001), and 2.70-fold (p < 0.001), and 7.60-fold (p < 0.001), respectively. In the case of hNP, the relative fold changes of protein secretion were 2.68-fold (p < 0.001), 10.09-fold (p < 0.001), 3.10-fold (p < 0.001), 2.52-fold (p < 0.01), 2.20-fold (p < 0.01), and 2.78-fold (p < 0.001), respectively. The original quantitative values without normalization are in Supplementary Tables S1–S4.

### Role of hResistin stimulation-induced upregulation of IL-1β, p38 MAPK, and p65 NF-κB and the combined hResistin and IL-1β stimulation-induced IL-6, IL-8, MMP-1, MMP-3, and MMP-13 changes via stimulation with IL-1β, p38 MAPK, and p65 NF-κB specific inhibitors

To confirm the pro-inflammatory cytokine activation nature of hResistin, the effects of IL-1β, p38 MAPK, and p65 NF-κB on the upregulation of inflammatory mediators induced by the combined-hResistin and IL-1β stimulation and the reduction in secretion of inflammatory mediators induced by the combined-hResistin and IL-1β stimulation and attributed to the treatment with IL-1, p38 MAPK, and p65 NF-κB-specific inhibitors IL-1Ra, SB203580, and BAY 11-7082, respectively, were analyzed compared to those observed upon the combined-hResistin and IL-1β or mono-IL-1β stimulation. As shown in Fig. 6, a clear trend between p65 NF-κB and the upregulation of combined-hResistin and IL-1β stimulation induced inflammatory mediators was observed via a significant reduction attributed to the treatment with BAY 11-7082 in both hAF and hNP cells. When correlating IL-1Ra treatment and ECM catabolic enzymes, the inhibition of ECM catabolic enzymes in NP cells was markedly higher than that in hAF cells. In hAF cells, the inhibition levels of mono-IL-1β in the IL-1Ra treatment group were higher than those in the IL-1Ra treatment group (Fig. 6, (I) IL-1Ra treatment panels). In the correlation between SB203580 treatment and the inflammatory mediators, increased inflammatory mediator levels in the combined groups tended to decrease almost to the same levels as those observed in the mono-IL-1β stimulation groups (Fig. 6, (II) SB203580 treatment panels). In addition, mono- IL-1β stimulation with SB203580 treatment showed a higher tendency to decrease than combined stimulation with SB203580 treatment in both hAF and hNP cells. Regarding IL-1Ra treatment, the inflammatory cytokine levels were similar to those obtained with BAY 11-7082 treatment, and the inflammatory cytokines were inhibited at higher levels than those in the mono-IL-1β stimulation with IL-1Ra treatment in both hAF and hNP cells (Fig. 6, (III) BAY 11-7082 treatment panels). The original quantitative values without normalization are in Supplementary Tables S2–S4.

## Discussion

Although several studies investigated the correlation between hResistin and the pro-inflammatory signaling pathways, the main cause of the promoting effect of hResistin on the promotion of IL-1β-associated pro-inflammatory cytokine cascades is still not fully understood. However, it is suspected that the activation of toll-like receptor (TLR)4, which is reported to serve as a receptor for the pro-inflammatory effects of resistin in human cells, could trigger the upregulation of not only the IL-1 family of cytokines but also of TNF-α^10,18–20^. As shown in Fig. 3, hResistin stimulation of both hAF and hNP cells induced the phosphorylation of p38 MAPK and p65 NF-κB, which promote inflammatory cytokine activation cascades in hIVD tissues. MAPK is activated by various exogenous factors and is involved in various inflammatory diseases, such as osteoarthritis, rheumatoid arthritis, and neurodegenerative disease^21,22^. MAPKs are divided into three subunit types: p38, ERK, and JNK. Regarding the inflammatory cytokines of the hIVD, p38 and ERK are associated with a decrease in ECM synthesis, and p38 and JNK are involved in the activation of ECM catabolic enzymes, such as MMPs^23,24^. Another important pathway involved in the inflammatory response involves NF-κB, a central component of the hIVD cellular response to damage, stress, and inflammation. NF-κB is composed of the most common heterodimer, the p50/p65 subunit complex, which regulates several pro-inflammatory mediators, such as TNF-α, IL-1β, IL-6, and MMPs^25,26^. Thus, p38 MAPK and p65 NF-κB may play important roles in inflammatory IVD degeneration. The phosphorylation of p38 MAPK and p65 NF-κB is associated with TLR4-related inflammasome upregulation^27–29^. The interaction between hResistin and hIVD cells results in the mRNA expression of NLRP3, Caspase-1, and pro-IL-1β. The inflammasome components involved in the inflammatory response are shown in Fig. 4. The gene expression of hResistin-induced inflammasome components exerts a greater influence in hNP cells, and the role of hResistin in the inflammatory response is expected to be more dominant in hNP cells than in hAF cells. Nevertheless, the gene expression induced by hResistin stimulation was significantly different from that of the unstimulated control groups, but the fold-change value was not significantly different; thus, hResistin mono-stimulation did not seem to significantly affect inflammatory cytokine expression in hIVD cells.

**Figure 5:**
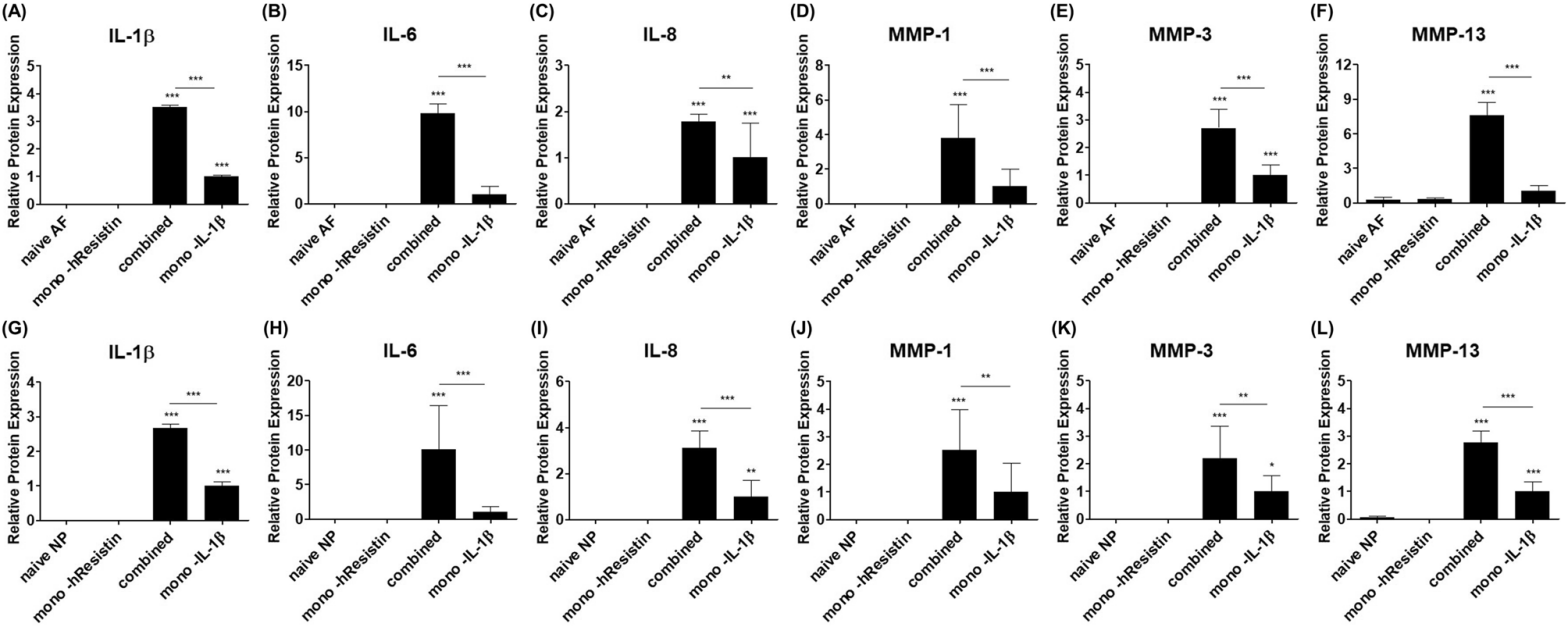
The change in secretion of (**A**) IL-1β, (**B**) IL-6, (**C**) IL-8, (**D**) MMP-1, (**E**) MMP-3, and (**F**) MMP-13 induced by hAF and hNP cells stimulated with mono-hResistin, combined-hResistin and IL-1β or mono-IL-1β for 48 h with respect to that of naïve group (*p < 0.05, **p < 0.005, ***p < 0.001, n = 7/group).

**Figure 6:**
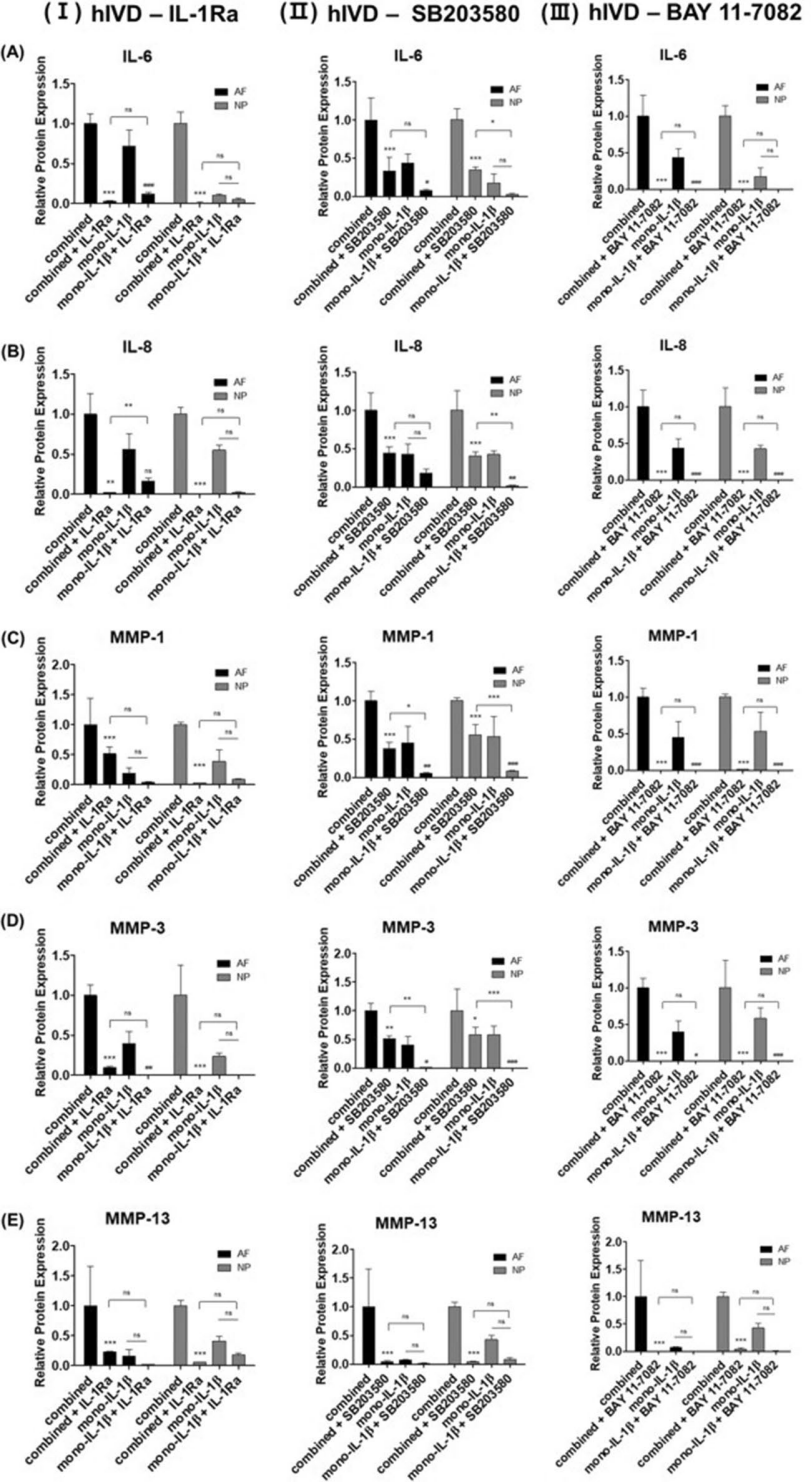
Combined-hResistin and IL-1β stimulation induced changes in the expression of secreted inflammatory cytokines and ECM catabolic enzymes (*p < 0.05, **p < 0.01, and ***p < 0.001 compared to the combined stimulation group, ^#^p < 0.05, ^##^p < 0.01, and ^###^p < 0.001, compared to the mono-IL1β stimulation group, n = 5/group) (**A**) IL-6, (**B**) IL-8, (**C**) MMP-1, (**D**) MMP-3, and (**E**) MMP-13 in hAF and hNP cells, followed by subsequent stimulation with IL-1, p38 MAPK, and p65 NF-κB-specific inhibitors, which are (I) IL-1Ra, (II) SB203580, and (III) BAY 11-7082.

Based on the significant increase in secretion of IL-1β, IL-6, IL-8, MMP-1, MMP-3, and MMP-13 induced by combined-hResistin and IL-1β stimulation of both hAF and hNP cells, compared with that induced by IL-1β mono-stimulation (Fig. 5), the promoting effect of hResistin on the activation of the IL-1β-associated pro-inflammatory cytokine cascade was observed. This cascade is associated with the progression of obesity-related inflammatory disc degeneration; this further supports the low-grade pro-inflammatory cytokine cascade activation nature of hResistin, which is widely reported to be associated with the development of insulin resistance in obesity^1,30–34^. hResistin is primarily expressed in immune cells, such as monocytic cells, which are key players in the inflammatory response, in contrast to rodent resistin, which releases rodent adipocytes in white adipose tissues^19,20,35^. Thus, hResistin is expected to have a greater influence on the inflammatory response in adipokine-related diseases than rodent resistin. The promoting effect of hResistin on the IL-1β-associated pro-inflammatory cytokine cascade might be influenced by the correlation between TLR4 and hResistin-induced expression of IL-1β and the phosphorylation of p38 MAPK and p65 NF-κB. The treatment with each specific inhibitor of IL-1β, p38 MAPK, and p65 NF-κB effectively inhibited the promoting effect of hResistin-related inflammatory mediator protein expression (Fig. 6). The IL-1 signaling inhibition of the combined groups reduced both the increased inflammatory cytokine and ECM catabolic enzyme expression levels, which may be due to a promoting effect of hResistin-induced low-grade inflammatory cytokine activation in the IL-1β-associated inflammatory response. In addition, the reduction of inflammatory cytokines and ECM catabolic enzymes in hAF cells were lower than in hNP cells, suggesting that hNP could play a more important role in disc degeneration than in hAF^36^. When comparing p38 MAPK and p65 NF-κB, which are involved in the expression of inflammatory mediators in hIVDD, the NF-κB signaling inhibition showed a greater reduction in levels of inflammatory mediators than p38 MAPK signaling inhibition, indicating that NF-κB signaling plays a more important role than p38 MAPK signaling. It is also thought that pathways other than the p38 MAPK pathway may be involved in ECM catabolic enzyme expression^37,38^. In 2014, Eltom et al. reported the release of IL-1β, induced by TLR4, which was activated via direct exposure to endotoxin lipopolysaccharide (LPS) in living mouse lung tissues in vivo^38,39^. Similarly, in the following year, Lin et al. also reported an increased expression of IL-1β and TNF-α induced by the activation of TLR4, which was induced by in vitro stimulation of synovial fibroblast cells with LPS^39,40^. It is widely accepted that IVDD is evident by the increased secretion of TNF, IL‑1α, IL‑1β, IL‑6, IL‑17, IL‑8, and IL‑2, which leads to an imbalance in catabolic and anabolic responses, leading to the degeneration of IVD tissues, as well as disc herniation and radicular pain via the promotion of ECM degradation, chemokine secretion, and IVD cell phenotype changes^41–43^. Furthermore, increases in the expression of MMP‑1, MMP‑3, MMP‑7, MMP‑9, and MMP‑13 are associated with the promotion of a degenerative response^38,44–46^. In 2005 and 2007, Le Maitre et al. reported that IL-1 agonist, IL-1Ra, IL-1R1, and IL-1-converting enzyme were produced in hIVD cells, and the remaining proteins increased except IL-1Ra along with severity of degeneration^12^. Also, by reporting an increase in the gene expression of IL-1 receptor type I rather than TNF receptor type I in degenerative IVD, the importance of IL-1 in disc degeneration was emphasized^47^. Our results also showed that when IL-1β was treated on IVD cells, the production of inflammatory mediators and ECM-catabolic enzymes was increased, and a statistically significant decrease was seen through IL-1Ra treatment. In another study, it was confirmed that IL-1β inhibits the expression of terminal complement complex (TCC), which plays a broad role in innate immunity, in AF, and that NP increases the deposition of TCC by the lysosomal protease cathepsin D^48^. These results are thought to cause differences in the responses of AF and NP in disc degeneration. Similar to our results, Gonçalves et al. in 2022 showed no significant change in hAF with single stimulation of a physiological cyclic tensile strain (CTS), but combined stimulation of CTS and IL-1β resulted in gene expression of inflammatory cytokines and ECM-catabolic enzymes^49^. This suggests that inflammatory cytokines contribute to IVDD phenotypes rather than physical factors, and our combined stimulation results of hResistin and IL-1β can be interpreted as results from adipokines and inflammatory cytokines in obesity. However, the specific inflammatory disc degeneration-associated inflammatory pathways that are responsible for the promoting effect of hResistin on the promotion of IL-1β-associated inflammatory cytokine cascade activation remain to be identified and should be investigated in future studies. Additionally, it was interesting to observe that the changes in secretion levels of all inflammatory mediators in hNP cells induced by the combined-hResistin and IL-1β stimulation were determined to be marginally greater than those induced in hAF cells. This trend may be due to the accelerated dehydration and desiccation of NP cells, induced by the promotion of apoptosis of NP cells activated by the IL family of inflammatory cytokines. Wang et al. reported that IL-2, which is associated with the onset of thinning and tearing of hAF cells at the early stage of inflammatory IVDD, promotes the apoptosis of NP cells by upregulating destructive enzymes, such as MMPs or ADAMTS, downregulating the aggrecan expression levels, and altering the levels of collagen type I–II via the death receptor pathway activated by an increased activity of caspase-3 and caspase-8, which is associated with the upregulation of Fas protein expression^50^. The NP is encapsulated by endplates and the AF has a significant influence on the overall function and homeostasis of the intervertebral discs by acting as a pump to regulate the flow of liquids and gases in the discs via proteoglycan‑rich ECM, maintaining the hydration levels^51^.

There is adipokine that acts on pro-inflammation like Resistin, while some adipokine that acts as an anti-inflammation like Adiponectin also exists. Adiponectin has anti-atherogenic and anti-inflammatory properties, and serum levels of Adiponectin tend to decrease from obesity^52,53^. In IVD tissue, adipoR1 and adipoR2 are widely expressed in hAF and hNP, and it has been reported that TNF-a and IL-6 gene expression are reduced during IL-1β treatment^54^. However, in IVDD, it has been reported that hNP decreases the level of adiponectin^55^. These results may suggest that adiponectin plays a significant role in anti-inflammation in maintaining homeostasis in disc environments in normal IVD, but Adiponectin levels decrease in obesity-accompanied IVDD levels and increase in Resistin levels affect inflammation and support our results.

**Figure 7:**
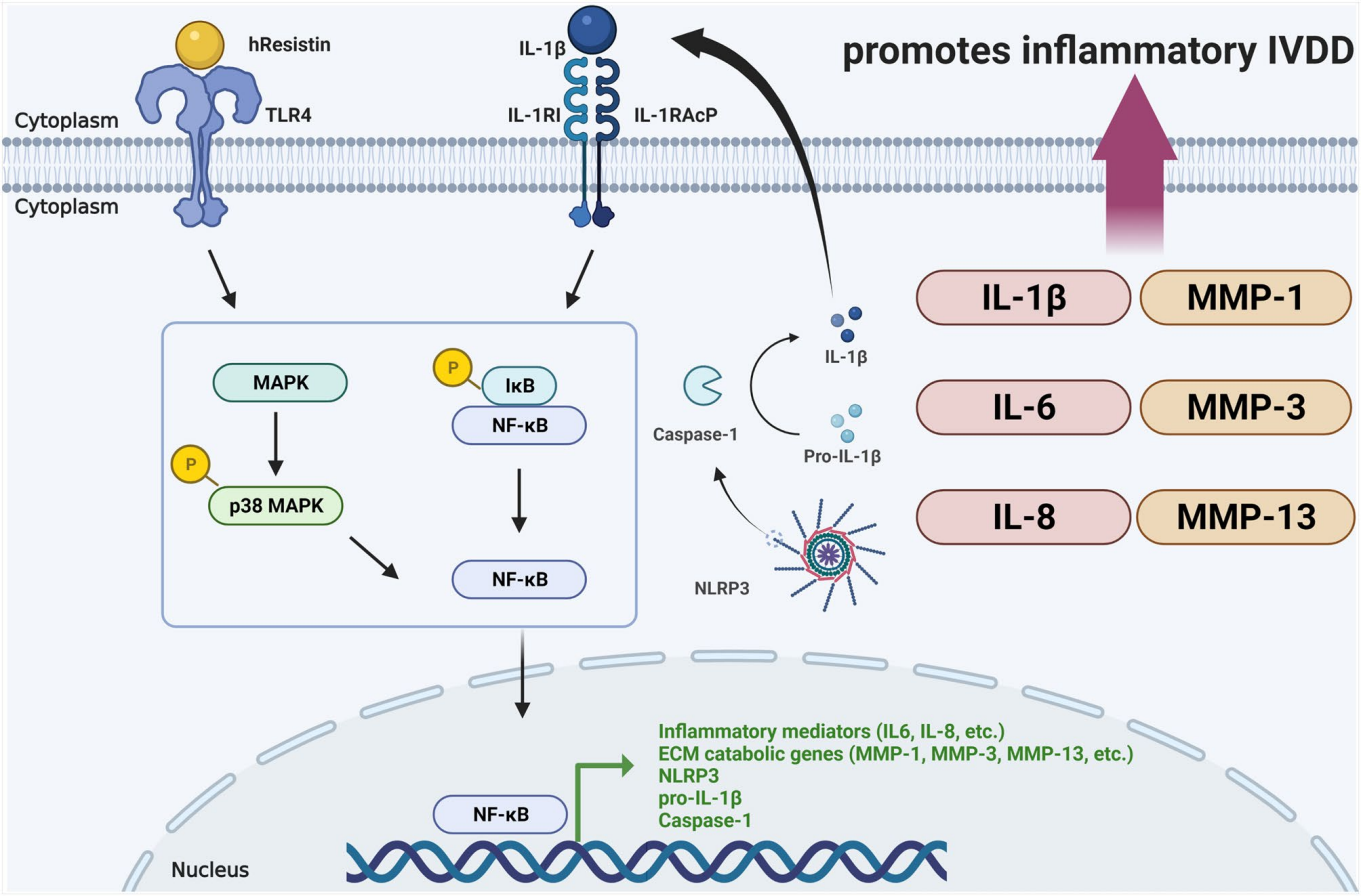
Schematic summary of the inflammatory response-promoting effect of hResistin in inflammatory IVDD. Resistin activates NF-κB and p38 MAPK through TLR4 in IVD cells to express NLRP3, pro-IL-1β and caspase-1. Pro-inflammatory IL-1β induces an inflammatory response through IL-1RI. In this process, hResistin promotes the production and secretion of inflammatory cytokines of IL-1β, IL-6, and IL-8 and ECM-catabolic enzymes of MMP-1, MMP-3, and MMP-13 through the IL-1β pathway.

In conclusion, based on the significant upregulation of target inflammatory IVDD-associated inflammatory mediators, induced by the combined-hResistin and IL-1β stimulation of hIVD cells, compared to that induced by mono-IL-1β stimulation, the promoting effect of hResistin, which is associated with the development of insulin resistance in obesity, on the progression of inflammatory IVDD via upregulation of inflammatory mediators associated with the activation of IL-1β pro-inflammatory cytokine cascade has been observed (Fig. 7). These results further support the low-grade pro-inflammatory cytokine cascade activation nature of hResistin. Moreover, it is suggested that the reduction in the production of inflammatory cytokines and ECM-catabolic enzymes, especially the signal block of NF-κB, may be an effective treatment method for hResistin-promoted inflammatory IVDD. The results of our study improve our understanding of the pro-inflammatory mechanism of action of hResistin, which is associated not only with the progression of inflammatory IVDD but also with other obesity- and diabetes-related inflammatory diseases.

## Supplementary Information

Supplementary Information.

## Data Availability

The datasets generated and/or analysed during the current study are available from the corresponding author upon reasonable request.
