# Supplementary material for: Adipokine human Resistin promotes obesity-associated inflammatory intervertebral disc degeneration via pro-inflammatory cytokine cascade activation
Source: Sci Rep. 2022 May 27;12:8936. doi: 10.1038/s41598-022-12793-2 (PMC9142523; doi:10.1038/s41598-022-12793-2)
Supplement: Supplementary file 1 — Supplementary Information. [file 41598_2022_12793_MOESM1_ESM.pdf]

Supplementary information for "**Adipokine human Resistin promotes obesity-associated inflammatory intervertebral disc degeneration via pro-inflammatory cytokine cascade activation**"

Jae Hee Shin[1,5], SeongHyun Park[2], Hansang Cho[3,5,6], Joo Han Kim[4] and Hyuk Choi[1]*

[1]*Department of Medical Sciences, Graduate School of Medicine, Korea University, Seoul, South Korea*

[2]*Department of Electrical and Electronic Engineering, The University of Manchester, Manchester, UK*

[3]*Department of Biophysics, Sungkyunkwan University, Suwon, South Korea*

[4]*Department of Neurosurgery, Guro Hospital, College of Medicine, Korea University, Seoul, South Korea*

[5]*Institute of Quantum Biophysics, Sungkyunkwan University, Suwon, South Korea*

[6]*Intelligent Precision Healthcare Convergence, Sungkyunkwan University, Suwon, South Korea*

Figure S1

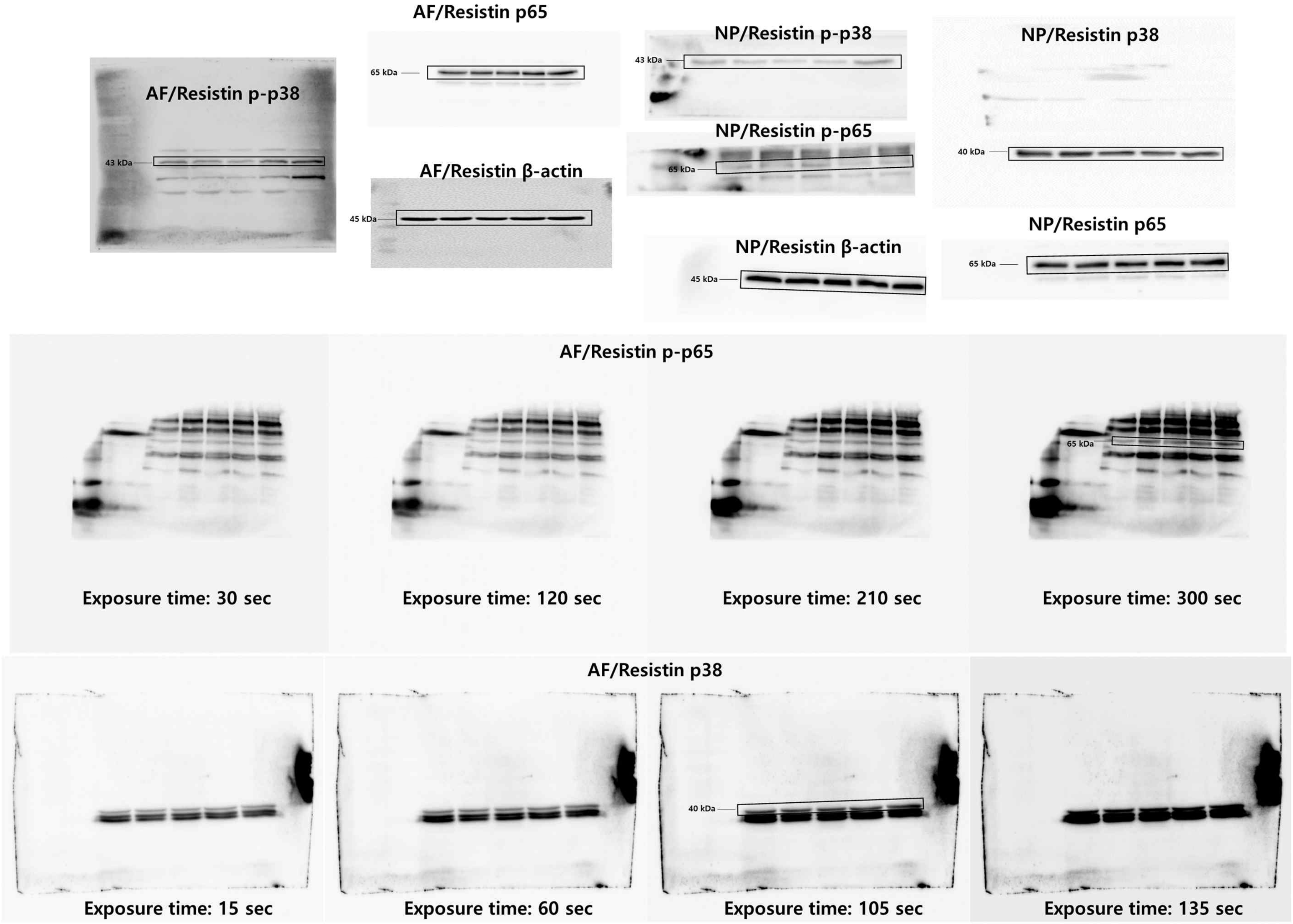

Figure S1. Correlation between p38 MAPK and p65 NF-κB for hResistin stimulation by several time-point in hIVD cells

Table S1.The change in secretion of inflammatory mediators (mean ± SD, unit: ng/mL)

	Naïve hAF	Mono-hResistin	combined	Mono-IL-1β	Naïve hNP	Mono-hResistin	combined	Mono-IL-1β
**IL-1β**	undetermined	undetermined	0.60 ± 0.01	0.17 ± 0.01	undetermined	undetermined	0.64 ± 0.02	0.17 ± 0.01
**IL-6**	undetermined	undetermined	7.47 ± 0.83	0.77 ± 0.69	undetermined	undetermined	7.54 ± 4.72	0.77 ± 0.69
**IL-8**	undetermined	undetermined	9.96 ± 0.98	5.62 ± 4.17	undetermined	undetermined	27.43 ± 6.58	5.62 ± 4.17
**MMP-1**	undetermined	undetermined	4.49 ± 2.35	1.19 ± 1.18	undetermined	undetermined	3.60 ± 2.07	1.19 ± 1.18
**MMP-3**	undetermined	undetermined	191.48 ± 48.41	70.87 ± 26.71	0.09 ± 0.03	0.33 ± 0.06	254.40 ± 133.99	70.87 ± 26.71
**MMP-13**	0.01 ± 0.01	0.02 ± 0.01	0.44 ± 0.06	0.44 ± 0.06	0.01 ± 0.01	undetermined	0.57 ± 0.08	0.06 ± 0.03

Table S2. The change in secretion of inflammatory mediators with or without IL-1Ra (mean ± SD, unit: ng/mL)

	**hAF combined**	**hAF combined with IL-1Ra**	**hAF Mono-IL-1β**	**hAF Mono-IL-1β with IL-1Ra**	**hNP combined**	**hNP combined with IL-1Ra**	**hNP mono-IL-1β**	**hNP mono-IL-1β with IL-1Ra**
**IL-6**	15.96 ± 1.91	0.50 ± 0.09	11.42 ± 3.24	1.96 ± 0.27	12.55 ± 1.84	0.09 ± 0.19	1.34 ± 0.21	0.62 ± 0.21
**IL-8**	11.28 ± 2.86	0.21 ± 0.06	6.36 ± 2.19	1.86 ± 0.44	23.20 ± 2.06	undetermined	12.77 ± 1.40	0.50 ± 0.26
**MMP-1**	14.85 ± 6.47	7.67 ± 1.73	2.89 ± 1.34	0.63 ± 0.06	6.44 ± 0.27	0.18 ± 0.04	2.50 ± 1.25	0.56 ± 0.06
**MMP-3**	302.01 ± 40.02	28.73 ± 5.01	118.97 ± 46.57	2.92 ± 0.14	340.36 ± 127.56	2.19 ± 0.41	81.23 ± 13.13	0.84 ± 0.08
**MMP-13**	1.75 ± 1.15	0.40 ± 0.02	0.27 ± 0.20	0.03 ± 0.01	0.63 ± 0.05	0.03 ± 0.001	0.26 ± 0.05	0.11 ± 0.02

Table S3. The change in secretion of inflammatory mediators with or without SB203580 (mean ± SD, unit: ng/mL)

	**hAF combined**	**hAF combined with SB203580**	**hAF Mono-IL-1β**	**hAF Mono-IL-1β with SB203580**	**hNP combined**	**hNP combined with SB203580**	**hNP mono-IL-1β**	**hNP mono-IL-1β with SB203580**
**IL-6**	26.27 ± 7.61	8.66 ± 4.68	11.42 ± 3.24	1.99 ± 0.42	12.55 ± 1.84	4.35 ± 0.47	2.19 ± 1.52	0.33 ± 0.15
**IL-8**	29.50 ± 6.69	12.98 ± 2.53	12.73 ± 3.95	5.36 ± 1.62	30.32 ± 7.80	12.35 ± 1.46	12.89 ± 1.46	0.55 ± 0.21
**MMP-1**	18.08 ± 2.28	6.76 ± 1.54	8.13 ± 3.96	1.00 ± 0.07	6.44 ± 0.27	3.58 ± 0.85	3.45 ± 1.67	0.54 ± 0.03
**MMP-3**	301.97 ± 40.07	124.12 ± 14.39	120.22 ± 45.52	2.95 ± 0.29	340.36 ± 127.56	198.36 ± 44.51	197.94 ± 49.72	0.78 ± 0.26
**MMP-13**	1.75 ± 1.15	0.07 ± 0.04	0.12 ± 0.02	0.03 ± 0.002	0.61 ± 0.05	0.03 ± 0.01	0.26 ± 0.05	0.05 ± 0.02

Table S4. The change in secretion of inflammatory mediators with or without BAY 11-7082 (mean ± SD, unit: ng/mL)

	**hAF combined**	**hAF combined with BAY 11-7082**	**hAF Mono-IL-1β**	**hAF Mono-IL-1β with BAY 11-7082**	**hNP combined**	**hNP combined with BAY 11-7082**	**hNP mono-IL-1β**	**hNP mono-IL-1β with BAY 11-7082**
**IL-6**	26.27 ± 7.61	undetermined	11.42 ± 3.24	undetermined	12.55 ± 1.84	undetermined	2.19 ± 1.52	undetermined
**IL-8**	29.50 ± 6.69	0.01 ± 0.01	12.74 ± 3.95	undetermined	30.32 ± 7.80	undetermined	12.89 ± 1.46	undetermined
**MMP-1**	18.08 ± 2.28	0.03 ± 0.04	8.13 ± 3.96	undetermined	6.44 ± 0.27	0.11 ± 0.01	3.45 ± 1.67	undetermined
**MMP-3**	301.97 ± 40.07	0.43 ± 0.42	120.22 ± 45.52	undetermined	340.36 ± 127.56	1.07 ± 0.15	197.94 ± 49.72	undetermined
**MMP-13**	1.75 ± 1.15	0.003 ± 0.004	0.12 ± 0.02	0.003 ± 0.004	0.61 ± 0.05	0.03 ± 0.01	0.26 ± 0.05	0.002 ± 0.002
